# Stress increases the risk of type 2 diabetes onset in women: A 12-year longitudinal study using causal modelling

**DOI:** 10.1371/journal.pone.0172126

**Published:** 2017-02-21

**Authors:** Melissa L. Harris, Christopher Oldmeadow, Alexis Hure, Judy Luu, Deborah Loxton, John Attia

**Affiliations:** 1 Research Centre for Generational Health and Ageing, Faculty of Health and Medicine, University of Newcastle, Newcastle, New South Wales, Australia; 2 Clinical Research Design, IT and Statistical Support Unit, Hunter Medical Research Institute, Newcastle, New South Wales, Australia; 3 School of Medicine and Public Health, Faculty of Health and Medicine, University of Newcastle, Newcastle, New South Wales, Australia; 4 Division of Medicine, John Hunter Hospital, Hunter New England Health, Newcastle, New South Wales, Australia; Garvan Institute of Medical Research, AUSTRALIA

## Abstract

**Background:**

Type 2 diabetes is associated with significant morbidity and mortality. Modifiable risk factors have been found to contribute up to 60% of type 2 diabetes risk. However, type 2 diabetes continues to rise despite implementation of interventions based on traditional risk factors. There is a clear need to identify additional risk factors for chronic disease prevention. The aim of this study was to examine the relationship between perceived stress and type 2 diabetes onset, and partition the estimates into direct and indirect effects.

**Methods and findings:**

Women born in 1946–1951 (n = 12,844) completed surveys for the Australian Longitudinal Study on Women’s Health in 1998, 2001, 2004, 2007 and 2010. The total causal effect was estimated using logistic regression and marginal structural modelling. Controlled direct effects were estimated through conditioning in the regression model. A graded association was found between perceived stress and all mediators in the multivariate time lag analyses. A significant association was found between hypertension, as well as physical activity and body mass index, and diabetes, but not smoking or diet quality. Moderate/high stress levels were associated with a 2.3-fold increase in the odds of diabetes three years later, for the total estimated effect. Results were only slightly attenuated when the direct and indirect effects of perceived stress on diabetes were partitioned, with the mediators only explaining 10–20% of the excess variation in diabetes.

**Conclusions:**

Perceived stress is a strong risk factor for type 2 diabetes. The majority of the effect estimate of stress on diabetes risk is not mediated by the traditional risk factors of hypertension, physical activity, smoking, diet quality, and body mass index. This gives a new pathway for diabetes prevention trials and clinical practice.

## Introduction

Diabetes is associated with significant morbidity and mortality, and contributes substantially to healthcare expenditure. Diabetes is projected to affect 552 million people by 2030, almost tripling in prevalence since 2011 [1]. Type 2 diabetes, characterised by insulin resistance, accounts for approximately 96% of all diabetes cases in adults aged over 25 [2]. It has been estimated that as much as 60% of type 2 diabetes disease risk is due to modifiable environmental factors including obesity, physical inactivity, diet quality, smoking, hypertension and abnormal cholesterol levels [3]. Despite the implementation of interventions based on these traditional risk factors, the incidence of diabetes continues to rise. Identifying additional factors that contribute to increased risk is of public health significance.

Increasingly, psychological stress is being explored as a risk factor for chronic conditions, such as cardiovascular disease and arthritis [4]. Diabetes and cardiovascular disease share some of the same causal pathways, hence there is logic in testing the relationship between stress and diabetes. Pathophysiological mechanisms linking stress to diabetes have included direct neuroendocrine effects (e.g. the fact that stress hormones such as cortisol and adrenaline are counter-regulatory to insulin), and indirect effects mediated by traditional risk factors (e.g. stress may reduce the likelihood of exercising) [5, 6].

When psychological stress, particularly work stress, has been examined as a risk factor for type 2 diabetes in epidemiological research, the findings have been equivocal [7, 8]. A Swedish study of men born 1921–1925 found that chronic stress (related to home and work) for one to five years prior to baseline was associated with a 45% increase in the risk of being diagnosed with either type 1 or type 2 diabetes (assessed using hospital records) at 35 year follow-up [9]. Likewise, Kato *et al.[10]* found that baseline perceived stress was associated with an increased risk of diabetes onset at ten-year follow-up in a Japanese cohort of men and women aged 40–69 years. On the other hand, an Australian study of men and women aged 25 years and over at baseline, recently found that high levels of perceived stress (as opposed to stressful life events) were associated with abnormal glucose metabolism over a five year period for women but not men [6]. Therefore, the way in which stress is measured, in particular the timing of exposure and the error involved, may be pivotal to understanding the stress and diabetes relationship. To our knowledge, no studies have examined the temporal relationship between perceived stress and type 2 diabetes, using repeated measures of stress. We account for the variation in perceived stress, as well as traditional risk factors, over time and at a population level. This study aims to:

1. examine the relationship between perceived stress and type 2 diabetes onset in a broadly representative cohort of middle-aged women over a 12-year period;
2. partition the total effect estimate of stress on diabetes into direct and indirect effects using mediation analysis.

## Materials and methods

### Overview of study design

This study included data from the 1946–1951 cohort of the Australian Longitudinal Study on Women’s Health, a national population-based study of physical, psychological, environmental and economic factors in Australian women. Women were randomly sampled through the Medicare Australia database, except women from rural and remote areas sampled at twice the rate as those from urban areas to provide adequate statistical power for comparisons to be made by area. Through comparisons with national census data the cohort has been deemed largely representative of the population of women in this age group [11]. Written informed consent was obtained from each person at each survey. This project has ongoing clearance from the University of Queensland and University of Newcastle’s Human Research Ethics Committees.

### Participants

This analysis focused on women from the 1946–1951 cohort who completed surveys in 1998 (Survey 2), 2001 (Survey 3), 2004 (Survey 4), 2007 (Survey 5) and 2010 (Survey 6). Survey 2 was set as the baseline for consistency in variable measurement. Of the 13,715 women who responded to the initial invitation in 1996, 12,338 (90%) completed Survey 2 when aged 47–52 years, with 10,011 (73%) remaining in the cohort at Survey 6 (aged 59–64 years). The longitudinal analysis related to 12,844 (94% of the original cohort) women who provided at least one data point at either Survey 2, 3, 4, 5 or 6, and did not report type 1 diabetes (n = 17).

### Causal model expressed as a directed acyclic graph

**Fig 1:**
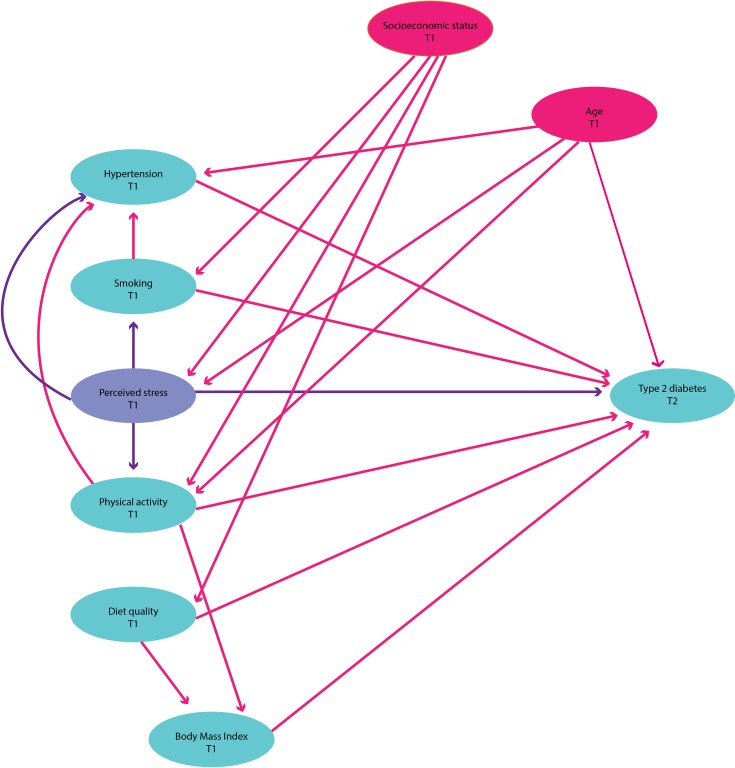
Simplified Directed Acyclic Graph showing hypothesised causal mechanism between perceived stress and type 2 diabetes taking into account potential confounders and mediators. According to the DAG, perceived stress (measured at Survey 2) may be mediated through physical activity, diet quality (or BMI instead of diet), hypertension and smoking status and confounded by age and socioeconomic status (i.e. highest educational qualification).

The causal model being tested in this study is articulated using the directed acyclic graph in Fig 1. The directed acyclic graph shows the main exposure (stress) and the main outcome (type 2 diabetes). Socioeconomic status and age both affect stress and diabetes and hence are considered confounders since they provide a “back-door path” between the outcome and the exposure. Perceived stress can influence smoking, hypertension, physical activity and body mass index, either directly or indirectly, all of which can then influence the risk of diabetes; these are therefore considered potential mediators.

Once the longitudinal nature of the study is taken into account, the causal web can get quite complicated (S1 Fig). In particular, the addition of the longitudinal dimension means that some mediators can now become confounders. In our case, stress at one point can influence physical activity at the next point, which in turn can influence stress again (visualised in S2 Fig). Logistic regression models cannot adequately handle this situation where a variable can be both a confounder (in which case it must be adjusted for) and a mediator (in which case adjusting for it would remove some of the effect we are trying to capture). For these cases, marginal structural models must be used.

### Measures

Unless otherwise stated, the following variables were measured at all surveys.

#### Outcome: Type 2 diabetes

Type 2 diabetes was defined as self-reported diagnosis or treatment for diabetes (excluding type 1) in the previous three years. Self-reported diabetes status has been found to be a reliable proxy for medical record review, particularly for women [12]. Impaired glucose tolerance is on the biological pathway to development of diabetes. Hence a sensitivity analysis was conducted where type 2 diabetes was defined as self-reported diagnosis/treatment for diabetes in the previous three years or diagnosis/treatment for impaired glucose tolerance at Surveys 3–6.

#### Predictor: Perceived stress

Women were asked to rate how stressed they had felt across ten life domains (including money and personal relationships) within a 12 month period on a five point scale from ‘not stressed at all’ to ‘extremely stressed’. Mean scores were aggregated into ‘no stress’ (mean score of 0), ‘minimal stress’ (scores >0 and ≤1) and ‘moderate/high stress’ (scores >1). This method of classification has been previously adopted to examine disease onset in other chronic conditions [4, 13]. This measure of perceived stress has demonstrated acceptable internal consistency (Cronbach’s alpha = 0.70) for the 1946–1951 cohort [14] and convergent and discriminant validity [15, 16]. For reference, Cronbach’s alpha coefficient for internal reliability was comparable with the commonly used 14-item Perceived Stress Scale (alpha = 0.75) [17].

#### Confounder variables

Age in years was determined from date of birth. Highest educational qualification was used as a measure of socioeconomic status.

#### Mediator variables

Hypertension was defined as self-reported diagnosis or treatment for the condition in the previous three years.

Physical activity was derived from Active Australia’s National Activity Survey (based on the frequency and duration of leisure-time activity lasting ten minutes or more in the last week). Weekly minutes were assigned a resting metabolic rate (MET) equivalent and were categorised as ‘no/low’ (<600 MET mins/week) and ‘moderate/high’ (≥600 MET mins/week) [18].

Diet quality was assessed at Survey 3 (2001) and Survey 5 (2007) using the Australian Recommended Food Score method [19], which allocates points for foods and beverages that are consistent with national dietary recommendations. Higher scores indicate better diet quality: higher dietary fibre, lower total and saturated fat, and higher micronutrient intakes [19]. For these analyses, diet scores were ranked as quintiles.

Smoking was categorised as ‘non-smoker’, ‘ex-smoker’ and ‘current smoker’ when examined as a predictor and as ‘non-smoker’, ‘ex-smoker/current smoker’ when examined as a mediator variable.

Body Mass Index (BMI) was used in sensitivity analyses instead of diet quantity. BMI was calculated for each participant from self-reported height and weight, categorised as: ‘underweight’ (<18·5m^2^), ‘healthy’ (18·5–24·99m^2^), ‘overweight’ (25–29·99m^2^) and ‘obese’ (≥30m^2^).

### Statistical analysis

Chi-square analyses (for categorical variables) and independent t-tests (for continuous variables) were used to examine sociodemographic differences according to type 2 diabetes status by Survey 6.

#### Estimating the total causal effects of perceived stress on type 2 diabetes

Associations between perceived stress and each of the hypothesised mediators (physical activity, diet quality, hypertension and smoking status) were examined using logistic regression (or ordinal logistic regression for diet quintiles), adjusting for potential confounders (age and education). Clustered robust variances were used to account for the repeated measurement on participants over time. Associations between each of the mediators and diabetes were then examined using separate pooled logistic regression models. Finally, the total causal effect estimate of perceived stress was examined using pooled logistic regression. Data were censored at type 2 diabetes diagnosis or the end of the observation period, whichever occurred first. Physical activity was identified as time varying: that is, depending on time it could be either affected by perceived stress (i.e. act as a mediator), or a confounder of the relationship between perceived stress and diabetes (S2 Fig). Therefore, a sensitivity analysis using marginal structural models to account for the effects of physical activity was performed. Marginal structural models were constructed with inverse probability of treatment weighting, where the weights were derived and used to circumvent the necessity to adjust for the time varying confounder. In short, inverse probability of treatment weighting “balances” the stress levels across prior physical activity levels such that the stress levels at each wave are not related to physical activity at previous waves. This is achieved by either up-weighting or down-weighting those with physical activity histories that are under-represented (or over-represented) in their current stress level.

#### Estimating the controlled direct effects of perceived stress on type 2 diabetes

Controlled direct effects were estimated by setting the mediator to a fixed level (through conditioning in the regression model), thereby controlling for the effects of perceived stress on the mediator. We calculated the fraction of the total effect explained by the mediators with 95% confidence intervals (95%CI) using a bootstrapping method (with 200 replications).

Models were constructed with and without a time lag (i.e. one survey or three years) at both a univariate and multivariate level (i.e. adjusting for confounders identified using a directed acyclic graph; see Fig 1 for a simplified model of hypothesised pathways and S1 Fig for the complete causal model). This method allowed for the examination of a temporal sequence (i.e. to allow for cause and effect) between perceived stress and type 2 diabetes onset, with psychological stress having to precede type 2 diabetes diagnosis [20].

As impaired glucose functioning is a key factor in type 2 diabetes [21], a sensitivity analysis was conducted with the diabetes category including women who reported either a diagnosis of glucose intolerance or type 2 diabetes. This approach accounted for women who may be symptomatic but had not yet been diagnosed with type 2 diabetes.

Statistical significance was set at p<0.05. All statistical analyse were conducted using Stata v13 (StataCorp, College Station, TX, USA).

## Results

### Sample characteristics

In 1998 (Survey 2; baseline in this study), 477 (3.7%) women reported a diagnosis of type 2 diabetes and were censored. By 2010 (Survey 6), 6.8% (n = 871) of women reported an incident diagnosis of type 2 diabetes. Sociodemographic characteristics for women with incident type 2 diabetes by Survey 6 compared to women without type 2 diabetes are shown in Table 1.

**Table 1:** Comparison of sociodemographic characteristics between women with incident type 2 diabetes compared with women without a diagnosis of type 2 diabetes over the observation period.

Characteristic	Type 2 Diabetes (n = 871)	No Type 2 Diabetes (n = 11,496)	*P* Value
Age (years, mean ± SD)	49.6 ± 1.5	49.5 ± 1.5	0.703
	n (%)	n (%)	
Marital status			
Partnered^a^	617 (73.5)	7,914 (77.9)	0.003
Unpartnered^b^	223 (26.6)	2,245 (22.1)	
Educational attainment^c^			
Tertiary/post graduate	87 (10.1)	1,682 (14.8)	<0.001
Trade/diploma	118 (13.7)	2,291 (20.1)	
School/ higher school certificate	424 (49.3)	5,545 (48.6)	
No formal	231 (26.9)	1,882 (16.5)	
Area of residence			
Major city	309 (37.0)	3,867 (38.2)	0.288
Inner regional	332 (39.7)	4,107 (40.6)	
Outer regional	160 (19.1)	1,832 (18.1)	
Remote/very remote	35 (4.2)	314 (3.1)	
Income management			
Impossible/difficult all the time	166 (21.0)	1,193 (12.4)	<0.001
Difficult some of the time	224 (28.3)	2,169 (22.6)	
Not too bad/easy	402 (50.8)	6,246 (65.0)	

^a^ Married or cohabitating

^b^ Separated, divorced, widowed or never married

^c^ Measured at Survey 1

### Direct and indirect effect estimates of stress on incident diabetes

Partitioning the effect estimates of stress on diabetes allows us to quantify the total effect, and divide this into an indirect effect, mediated through known variables, and a direct effect, which as the name implies, represents either a direct effect on diabetes or an effect through as yet unknown variables. We build up the model for the total effect of stress on diabetes by: first exploring the relationship between stress and known mediators; then the relationship between the known mediators and diabetes; and finally, by looking at the multivariate model.

#### Longitudinal associations between perceived stress and mediators

Table 2 shows the longitudinal relationship between perceived stress and the hypothesised mediators. As the models produced with and without a time lag were similar, only the results from the time lag models are reported here. A graded relationship was found between perceived stress and all mediators in the multivariate analyses. In particular, experiencing moderate/high levels of perceived stress was associated with a 1.61-fold increase in the odds ratio (OR) of having ever smoked (95%CI 1.42, 1.83; *P*<0.001), and a 1.67-fold increase in having been diagnosed or treated for hypertension (95%CI 1.46, 1.90; *P*<0.001). Further, women reporting moderate/high levels of perceived stress reported lower diet quality (OR 0.79, 95%CI 0.71, 0.88; *P*<0.001) and lower physical activity (OR 0.61, 95%CI 0.55, 0.68; *P*<0.001) compared to women with no stress.

**Table 2:** Longitudinal associations between perceived stress and hypothesised mediators using a time lag approach. Each analysis is adjusted for the potential confounders of SES (measured by educational attainment) and age, as well as secular trends (time by survey).

Variable	Stress → Diet		Stress → Smoking^a^		Stress → Hypertension		Stress → Physical activity	
	OR (95%CI)	P Value	OR (95%CI)	P Value	OR (95%CI)	P Value	OR (95%CI)	P Value
Perceived stress								
No stress	1.00		1.00		1.00		1.00	
Minimal stress	1.00 (0.91, 1.09)	0.930	1.25 (1.13, 1.38)	<0.001	1.25 (1.12, 1.39)	<0.001	0.76 (0.69, 0.82)	<0.001
Moderate/high stress	0.79 (0.71, 0.88)	<0.001	1.61 (1.42, 1.83)	<0.001	1.67 (1.46, 1.90)	<0.001	0.61 (0.55, 0.68)	<0.001
Educational attainment								
Tertiary/post graduate	1.00		1.00		1.00		1.00	
Trade/diploma	1.04 (0.95, 1.15)	0.417	1.23 (1.08, 1.40)	0.002	1.14 (1.00, 1.30)	0.060	0.91 (0.83, 1.00)	0.061
School/ higher school certificate	0.80 (0.73, 0.87)	<0.001	1.13 (1.00, 1.25)	0.038	1.32 (1.17, 1.48)	<0.001	0.75 (0.69, 0.81)	<0.001
No formal	0.53 (0.48, 0.59)	<0.001	1.54 (1.34, 1.77)	<0·001	1.47 (1.28, 1.69)	<0.001	0.51 (0.46, 0.57)	<0.001
Age (per year)	1.02 (1.00, 1.04)	0.034	0.99 (0.97, 1.02)	0.480	1.07 (1.04, 1.09)	<0.001	1.01 (0.99, 1.03)	0.576
Time (per survey)	0.94 (0.88, 1.00)	0.037	1.03 (0.96, 1.12)	0.412	1.01 (0.93, 1.09)	0.892	1.14 (1.07, 1.21)	<0.001

^a^As smoking status was treated as an outcome, for this analysis it was categorised as ‘non-smoker’ and ‘ex-smoker/smoker’.

#### Longitudinal associations between mediators and type 2 diabetes

In the time lagged multivariate analyses, a significant association with diabetes was found for both hypertension and physical activity (Table 3). Compared to women without hypertension, the presence of hypertension was associated with a 2.63-fold (95%CI 2.28, 3.04; *P*<0.001) increase in odds of reporting type 2 diabetes at the next survey (i.e. three years). Likewise, women who reported low levels of physical activity (compared to those with moderate or high levels) were 1·54 times more likely to report type 2 diabetes at the next survey (95%CI 1.33, 1.79; *P*<0.001). No significant lagged effect was found for either smoking status or diet on type 2 diabetes. However, in a sensitivity analysis using BMI instead of diet quality, an association was found for increasing BMI (S1 Table). In particular, compared to underweight women, being overweight was associated with a 2.49-fold (95%CI 1.98, 3.14; *P*<0.001) increase in odds of being diagnosed/treated for type 2 diabetes at the following survey, while those who were in the highest BMI category (obese) were 7.38 (95%CI 5.96, 9.13; *P*<0.001) times more likely to go on to develop type 2 diabetes.

**Table 3:** Longitudinal associations between the hypothesised mediators and type 2 diabetes, using a time lag approach. Each analysis is adjusted for the potential confounders of SES (measured by educational attainment) and age, as well as secular trends (time by survey). The aim here is to identify the relationship between each mediator and the outcome of type 2 diabetes. The combined effect of all the mediators is modelled in Table 4.

Variable	Diet → Type 2 Diabetes		Smoking → Type 2 Diabetes		Hypertension → Type 2 Diabetes		Physical activity → Type 2 Diabetes	
	OR (95%CI)	P Value	OR (95%CI)	P Value	OR (95%CI)	P Value	OR (95%CI)	P Value
Diet quintile (per unit)	0.96 (0.89, 1.03)	0.228						
Smoking status								
Non-smoker			1.00					
Ex-smoker			1.09 (0.94, 1.28)	0.262				
Smoker			1.14 (0.93, 1.41)	0.219				
Hypertension								
No					1.00			
Yes					2.63 (2.28, 3.04)	<0.001		
Physical activity								
Moderate/high							1.00	
None/low							1.54 (1.33, 1.79)	<0.001
Educational attainment								
Tertiary/post graduate	1.00		1.00		1.00		1.00	
Trade/diploma	1.15 (0.78, 1.70)	0.486	1.08 (0.81, 1.44)	0·593	1.05 (0.79, 1.40)	0.735	1.08 (0.81, 1.43)	0.612
School/ higher school certificate	1.46 (1.05, 2.05)	0.026	1.62 (1.27, 2.06)	<0.001	1.52 (1.20, 1.94)	0.001	1.58 (1.24, 2.01)	<0.001
No formal	2.60 (1.81, 3.76)	<0.001	2.68 (2.06, 3.49)	<0.001	2.51 (1.93, 3.26)	<0.001	2.53 (1.95, 3.30)	<0.001
Age (per year)	1.02 (0.95, 1.10)	0.523	1.04 (0.99, 1.09)	0.175	1.02 (0.97, 1.07)	0.439	1.04 (0.99, 1.09)	0.164
Time (per survey)	1.19 (0.94, 1.51)	0.150	1.22 (1.03, 1.44)	0.018	1.23 (1.04, 1.45)	0.014	1.23 (1.04, 1.45)	0.013

#### Total causal effect estimate of perceived stress on type 2 diabetes

A graded relationship for the total effect of perceived stress on type 2 diabetes onset was found (Table 4). In particular, women with minimal (OR 1.56, 95%CI 1.14, 2.14; *P* = 0.005) and moderate/high stress (OR 2.33, 95%CI 1.65, 3.28; *P*<0.001) demonstrated increases in odds of being diagnosed/treated for type 2 diabetes three years later, relative to no perceived stress. In a sensitivity analysis defining the outcome as either type 2 diabetes or glucose intolerance, the relationship increased slightly for those with moderate/high levels of perceived stress in the adjusted time lag analyses (S2 Table). The sensitivity analysis treating physical activity as a time varying confounder through marginal structural models had no influence on the results (S3 Table).

**Table 4:** Total causal effects of perceived stress on type 2 diabetes using a time lag approach (assuming physical activity as a time varying mediator).^a^^,^^b^

Variable	Model adjusted for confounders		Model adjusted for all mediators and confounders	
	OR (95%CI)	P Value	OR (95%CI)	P Value
Perceived stress				
None	1.00		1.00	
Minimal	1.56 (1.14, 2.14)	0.005	1.40 (1.01, 1.93)	0.043
Moderate/high	2.33 (1.65, 3.28)	<0.001	1.84 (1.29, 2.63)	0.001
Educational attainment				
Tertiary/post graduate	1.00		1.00	
Trade/diploma	1.09 (0.82, 1.45)	0.549	0.97 (0.72, 1.30)	0.816
School/ higher school certificate	1.65 (1.30, 2.10)	<0.001	1.32 (1.03, 1.69)	0·026
No formal	2.73 (2.11, 3.55)	<0.001	1.89 (1.44, 2.47)	<0.001
Age (per year)	1.04 (0.99, 1.09)	0.124	1.03 (0.98, 1.08)	0.314
Time (per survey)	1.22 (1.04, 1.43)	0.017	1.19 (1.01, 1.40)	0.041
Hypertension				
No			1.00	
Yes			1.82 (1.56, 2.12)	<0.001
Physical activity				
Moderate/high			1.00	
None/low			1.22 (1.05, 1.42)	0.010
BMI				
Underweight			1.00	
Healthy weight			2.29 (1.06, 4.96)	0.035
Overweight			2.25 (1.78, 2.84)	<0.001
Obese			5.88 (4.72, 7.32)	<0.001

^a^As smoking was not associated with diabetes onset, it was not included in this analysis

^b^As BMI was found to have a relationship with diabetes it was included instead of diet

#### Controlled direct effect of perceived stress on type 2 diabetes

When the controlled direct effect of perceived stress on type 2 diabetes onset was examined using traditional regression adjustment for all confounders and mediators, only slightly attenuated effects were observed (Table 4). Collectively, BMI, hypertension and physical activity explained only 10% (95%CI 3.5%, 18%) of the excess diabetes risk due to minimal perceived stress and 20% (95%CI 14%, 28%) of the variation due to moderate/high perceived stress. Stated conversely, about 80% of the effect of stress on increasing diabetes could not be explained simply by the influence on traditional risk factors (e.g. BMI, physical activity).

## Discussion

This study examined the temporal relationship between perceived stress and incident type 2 diabetes in a middle-aged cohort of Australian women over 12 years. Perceived stress was a strong risk factor for type 2 diabetes, with a graded relationship identified in the longitudinal time lag analyses. Our mediation analysis shows that only a small portion of the effect of stress on diabetes risk (i.e. <20%) is acting through traditional risk factors, particularly hypertension, BMI, and physical activity.

Our findings support and extend previous research examining the relationship between stress and diabetes onset, particularly among women. Williams *et al*. [6] found an independent effect of baseline perceived stress levels on incident impaired glucose metabolism in women over a five year period. Similarly, a graded relationship between post-traumatic stress disorder symptoms (an extreme example of chronic stress) and type 2 diabetes risk was shown using 22 years of data from the Nurses Health Study II [22].

Contrary to the long-standing ‘Bjorntorp hypothesis’, which proposes that neuroendocrine responses to stress are mediated mainly by central adiposity [23], BMI explained little of the variance in the perceived stress-diabetes relationship. Williams *et al*. [6] also showed adiposity (and health behaviours) had little influence on the relationship between stress and abnormal glucose metabolism. Likewise, Toshihiro and colleagues [24] found that obesity did not predict progression to diabetes from impaired fasting glucose and/or impaired glucose tolerance in Japanese workers.

In our study, we also found no relationship between smoking and type 2 diabetes onset over the 12 years. This is in sharp contrast with a 2007 meta-analysis of 25 prospective cohort studies [25]. It is important to note that the temporal relationship between smoking and type 2 diabetes in the studies that were pooled was not specifically explored and the studies included in the meta-analysis varied in terms of quality, follow-up and control of confounders. In particular, the findings of the pooled analyses appeared to be highly dependent on BMI, which raises the possibility that adjustment for BMI increases confounding of the stress/diabetes relationship, through collider bias [26]. Despite this, a significant relationship between moderate to high levels of perceived stress and traditional risk factors (e.g. diet and physical activity) was detected, as it has been in previous literature [27].

The large, controlled, direct effect of stress on diabetes may act on glucose metabolism through dysregulation of the hypothalamic-pituitary-adrenal and sympathetic-adrenal-medullary axes. The acute stress response activates the central, autonomic, neuroendocrine, and immune systems, as well as motor responses, when there are real or perceived threats to homeostasis [28]. In particular, the sympathetic-adrenal-medullary axis releases catecholamines and the hypothalamic-pituitary-adrenal axis secretes glucocorticoids that mobilise the ‘fight or flight’ response [29]. Chronic activation of the hypothalamic-pituitary-adrenal and sympathetic-adrenal-medullary axes and associated mechanisms, in conjunction with resultant amplification of pro-inflammatory cytokines (through increased tumor necrosis factor-α and interleukin-6 production), all counteract insulin and may induce insulin resistance and β-cell dysfunction [30, 31].

This study has a number of strengths. These include the prospective study design, large national cohort, use of sophisticated statistical techniques in order to examine mediational pathways, as well as the length of follow-up. In particular, the regularity of the survey data over the 12 years of follow-up allowed us to examine population-level changes in the predictor, mediators and confounders over time. To date no study has been able to achieve this.

The study must also be considered in light of its limitations. Firstly, we used a self-report measure of type 2 diabetes. However, self-reported diabetes status has been found to be a reliable proxy for medical record review, particularly for women [12]. It is possible that women with glucose intolerance were under-represented, since onset of diabetes may occur years prior to a clinical diagnosis [32]. However, the relationship between perceived stress and diabetes increased when including glucose intolerance in the sensitivity analysis. Secondly, in the lagged analyses diet quality was only measured twice, which may have contributed to its lack of prediction as a risk factor for type 2 diabetes. However, a recent meta-analysis of prospective cohort studies is in accord with the null finding [33] and BMI (which is related to dietary intake, and reported at each survey) was a predictor. Finally, given that both measures of stress and diabetes relied on self-report, it is possible they share common variance as a result of negative cognitive appraisal, which may increase the likelihood of detecting a significant association.

The findings of this study have important implications for the delivery of diabetes prevention and treatment programs. Current international consensus guidelines on the prevention of type 2 diabetes advocate for interventions administered at a whole population level as well as specific screening for high risk individuals [34]. This raises the possibility that measures of stress could improve the performance of current screening tools for adults at risk of type 2 diabetes, or that interventions to improve mental health may mitigate the growing incidence of type 2 diabetes. Targeting improved mental health and reducing stress could also have the potential of slowing the progression from glucose intolerance to frank diabetes, or lead to better glycaemic control. Importantly, a meta-analysis of randomised controlled trials of psychological interventions for type 2 diabetes found stress reduction improved long-term glycaemic control among type 2 diabetes patients [35]. In a complementary approach, moderate physical activity has been found to not only increase glucose utilisation in those with glucose intolerance [36] but also provides antidepressant effects [37].

With type 2 diabetes set to surpass cardiovascular disease as the number one chronic disease affecting society, addressing the increasing burden associated with type 2 diabetes will become a key healthcare priority. Using causal modeling techniques in this large national cohort study, perceived stress was found to be a strong independent risk factor for diabetes. The findings provide support for perceived stress to be considered alongside other modifiable risk factors for type 2 diabetes, such as obesity and physical activity in public health primary prevention and screening programs.

## Supporting information

S1 Fig Complete directed acyclic graph demonstrating hypothesised causal pathways between perceived stress and type 2 diabetes across the observation period. (DOC)

S2 Fig The role of physical activity in the relationship between perceived stress and type 2 diabetes. (DOC)

S1 Table Sensitivity analysis examining the longitudinal association between Body Mass Index (BMI) and type 2 diabetes, using a time lag approach. (DOC)

S2 Table Longitudinal models (with and without a time lag), reporting odds ratios and 95% confidence intervals (CI) for the relationship between perceived stress and type 2 diabetes/glucose intolerance (sensitivity analysis). (DOC)

S3 Table Sensitivity analysis comparing the total causal effect of stress on diabetes with physical activity treated as a time-varying confounder using a marginal structural model and a mediator using standard regression. (DOC)

## Abbreviations

BMI: body mass index
OR: odds ratio
95%CI: 95% confidence interval
