# Supplementary material for: Stress increases the risk of type 2 diabetes onset in women: A 12-year longitudinal study using causal modelling
Source: PLoS One. 2017 Feb 21;12(2):e0172126. doi: 10.1371/journal.pone.0172126 (PMC5319684; doi:10.1371/journal.pone.0172126)
Supplement: S1 Fig — (DOC) [file pone.0172126.s001.doc]

**
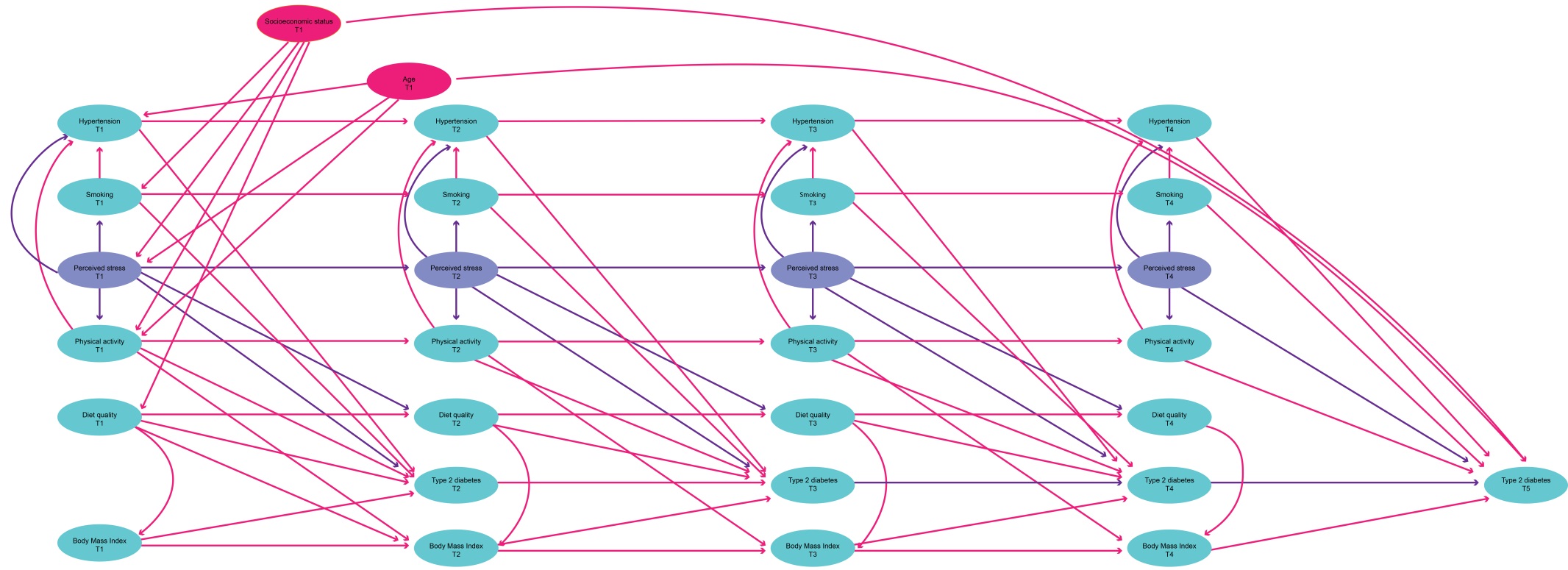
**

**S1 Figure. Complete directed acyclic graph demonstrating hypothesised causal pathways between perceived stress and type 2 diabetes across the observation period.**
