# Supplementary material for: Stress increases the risk of type 2 diabetes onset in women: A 12-year longitudinal study using causal modelling
Source: PLoS One. 2017 Feb 21;12(2):e0172126. doi: 10.1371/journal.pone.0172126 (PMC5319684; doi:10.1371/journal.pone.0172126)
Supplement: S2 Fig — (DOC) [file pone.0172126.s002.doc]

**
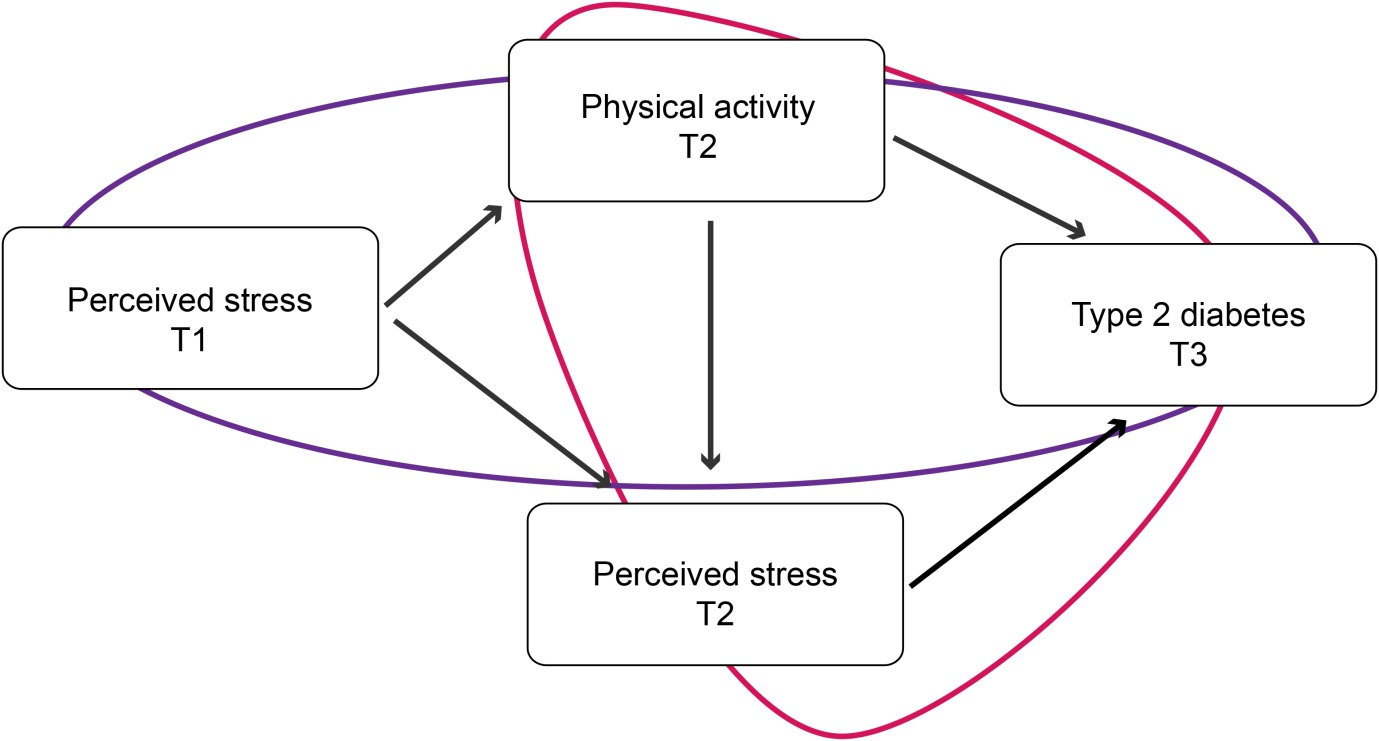
**

**S2 Figure. The role of physical activity in the relationship between perceived stress and type 2 diabetes.** The diagram shows the relationship between perceived stress and type 2 diabetes with physical activity acting as a time-varying confounder (shown in pink) and as a mediator (shown in purple).
