# Supplementary material for: Stress increases the risk of type 2 diabetes onset in women: A 12-year longitudinal study using causal modelling
Source: PLoS One. 2017 Feb 21;12(2):e0172126. doi: 10.1371/journal.pone.0172126 (PMC5319684; doi:10.1371/journal.pone.0172126)
Supplement: S1 Table — (DOC) [file pone.0172126.s003.doc]

**S1 Table. Sensitivity analysis examining the longitudinal association between Body Mass Index (BMI) and type 2 diabetes**, using a time lag approach.

| **Variable** | **BMI** 🡪 **Type 2 Diabetes** | |
| --- | --- | --- |
|  | **OR (95%CI)** | **P Value** |
| BMI |  |  |
| Underweight | 1.0 |  |
| Healthy weight | 2.3 (1.1, 4.9) | 0.037 |
| Overweight | 2.5 (2.0, 3.1) | <0.001 |
| Obese | 7.4 (6.0, 9.1) | <0.001 |
| Educational attainment |  |  |
| Tertiary/post graduate | 1.0 |  |
| Trade/diploma | 0.96 (0.72, 1.3) | 0.800 |
| School/ higher school certificate | 1.3 (1.0, 1.7) | 0.028 |
| No formal | 1.9 (1.5, 2.5) | <0.001 |
| Age (per year) | 1.0 (0.98, 1.1) | 0.242 |
| Time (per survey) | 1.2 (1.0, 1.4) | 0.044 |
