# Supplementary material for: Stress increases the risk of type 2 diabetes onset in women: A 12-year longitudinal study using causal modelling
Source: PLoS One. 2017 Feb 21;12(2):e0172126. doi: 10.1371/journal.pone.0172126 (PMC5319684; doi:10.1371/journal.pone.0172126)
Supplement: S2 Table — (DOC) [file pone.0172126.s004.doc]

**S2 Table.** Longitudinal models (with and without a time lag), reporting odds ratios and 95% confidence intervals (CI) for the relationship between perceived stress and type 2 diabetes/glucose intolerance (sensitivity analysis).

|  | **Without a time lag** | | | | **With a time lag** | | | |
| --- | --- | --- | --- | --- | --- | --- | --- | --- |
| **Variable** | **Unadjusted** | | **Adjusted** | | **Unadjusted** | | **Adjusted** | |
|  | **OR (95% CI)** | **P Value** | **OR (95%CI)** | **P Value** | **OR (95%CI)** | **P Value** | **OR (95%CI)** | **P Value** |
| Perceived stress |  |  |  |  |  |  |  |  |
| None | 1.0 |  | 1.0 |  | 1.0 |  | 1.0 |  |
| Minimal | 1.3 (1.1, 1.7) | 0.006 | 1.4 (1.2, 1.8) | 0.001 | 1.5 (1.2, 1.9) | <0.001 | 1.6 (1.3, 2.1) | <0.001 |
| Moderate/high | 2.1 (1.7, 2.6) | <0.001 | 2.2 (1.8, 2.8) | <0.001 | 2.3 (1.8, 3.0) | <0.001 | 2.5 (1.9, 3.2) | <0.001 |
| Educational attainment |  |  |  |  |  |  |  |  |
| Tertiary/post graduate |  |  | 1.0 |  |  |  | 1.0 |  |
| Trade/diploma |  |  | 1.1 (0.94, 1.4) | 0.185 |  |  | 1.1 (0.93, 1.4) | 0.219 |
| School/ higher school  certificate |  |  | 1.4 (1.2, 1.7) | <0.001 |  |  | 1.4 (1.2, 1.7) | <0.001 |
| No formal |  |  | 2.2 (1.8, 2.7) | <0.001 |  |  | 2.1 (1.8, 2.5) | <0.001 |
| Age (per year) |  |  | 1.0 (1.0, 1.1) | 0.020 |  |  | 1.1 (1.0, 1.1) | 0.011 |
