# Supplementary material for: Stress increases the risk of type 2 diabetes onset in women: A 12-year longitudinal study using causal modelling
Source: PLoS One. 2017 Feb 21;12(2):e0172126. doi: 10.1371/journal.pone.0172126 (PMC5319684; doi:10.1371/journal.pone.0172126)
Supplement: S3 Table — (DOC) [file pone.0172126.s005.doc]

**S3 Table. Sensitivity analysis comparing the total causal effect of stress on diabetes with physical activity treated as a time-varying confounder using a marginal structural model and a mediator using standard regression.**

|  | **Marginal Structural Model** | | | | **Standard Regression** | | | |
| --- | --- | --- | --- | --- | --- | --- | --- | --- |
| **Covariate** | **OR** | **Std. Err.** | **P value** | **95%CI** | **OR** | **Std. Err.** | **P value** | **95%CI** |
| Stress(lag1) |  |  |  |  |  |  |  |  |
| No/minimal* | 1 |  |  |  | 1 |  |  |  |
| Moderate/High | 1.564 | 0.151 | <0.001 | (1.294 to 1.889) | 1.547 | 0.142 | <0.001 | (1.292 to 1.852) |
| Age |  |  |  |  |  |  |  |  |
| per year | 1.028 | 0.027 | 0.289 | (0.977 to 1.082) | 1.037 | 0.026 | 0.141 | (0.988 to 1.089) |
| Educational attainment |  |  |  |  |  |  |  |  |
| tertiary/post graduate* | 1 |  |  |  | 1 |  |  |  |
| trade/diploma | 1.087 | 0.166 | 0.583 | (0.807 to 1.465) | 1.081 | 0.158 | 0.592 | (0.812 to 1.439) |
| school/HSC | 1.650 | 0.211 | <0.001 | (1.284 to 2.121) | 1.626 | 0.199 | <0.001 | (1.279 to 2.067) |
| no formal | 2.608 | 0.365 | <0.001 | (1.983 to 3.431) | 2.687 | 0.357 | <0.001 | (2.071 to 3.486) |
| Time |  |  |  |  |  |  |  |  |
| per wave | 1.235 | 0.108 | 0.016 | (1.040 to 1.466) | 1.219 | 0.100 | 0.015 | (1.038 to 1.431) |
